# Efficacy and safety of echinocandins versus triazoles for the prophylaxis and treatment of fungal infections: a meta-analysis of RCTs

**DOI:** 10.1007/s10096-014-2287-4

**Published:** 2014-12-14

**Authors:** J.-F. Wang, Y. Xue, X.-B. Zhu, H. Fan

**Affiliations:** Department of Pharmacy, Sir Run Run Shaw Hospital, School of Medicine, Zhejiang University, 3 East Qingchun Road, Hangzhou, 310016 Zhejiang People’s Republic of China

## Abstract

Echinocandins and triazoles were proven to be effective antifungal drugs against invasive fungal infections (IFI), which may cause significant morbidity and mortality in immunocompromised patients. The aim of this study was to compare the efficacy and safety between echinocandins and triazoles for the prophylaxis and treatment of fungal infections. PubMed, Embase, and the Cochrane Library were searched to identify relevant randomized controlled trials (RCTs) up to July 2014. The quality of trials was assessed with the Jadad scoring system. The primary outcomes of interest were treatment success, microbiological success, breakthrough infection, drug-related adverse events (AEs), withdrawals due to AEs, and all-cause mortality. Ten RCTs, involving 2,837 patients, were included, as follows: caspofungin versus fluconazole (*n* = 1), caspofungin versus itraconazole (*n* = 1), anidulafungin versus fluconazole (*n* = 1), micafungin versus fluconazole (*n* = 4), micafungin versus voriconazole (*n* = 2), and micafungin versus itraconazole (*n* = 1). Echinocandins and triazoles showed similar effects in terms of favorable treatment success rate [relative risk (RR) = 1.02, 95 % confidence interval (CI), 0.97–1.08], microbiological success rate (RR = 0.98, 95 % CI, 0.90–1.15), breakthrough infection (RR = 1.09; 95 % CI, 0.59–2.01), drug-related AEs (RR = 0.94; 95 % CI, 0.71–1.15), and all-cause mortality (RR = 0.85; 95 % CI, 0.66–1.10) in the prophylaxis and treatment of fungal infections. Additionally, echinocandins were more effective than triazoles for prophylaxis in patients undergoing hematologic malignancies or those who received hematopoietic stem cell transplantation (HSCT; RR = 1.08; 95 % CI, 1.02–1.15). Echinocandins significantly decreased the AE-related withdrawals rate compared with triazoles (RR = 0.47; 95 % CI, 0.33–0.67). This meta-analysis revealed that echinocandins are as effective and safe as triazoles for the prophylaxis and treatment of patients with fungal infections.

## Introduction

Invasive fungal infections (IFI) have emerged as a significant cause of morbidity and mortality in immunocompromised patients, particularly those with solid tumors or hematological malignancies [1–3], solid organ transplant [4], human immunodeficiency virus (HIV) infection [5, 6], and critical illness [7, 8]. *Candida albicans*, *Cryptococcus neoformans*, *Aspergillus fumigatus*, and *Pneumocystis jirovecii* are the most well-known causes of opportunistic mycosis [9]. The mortality attributable to candidemia is in the range 30–50 % [10, 11], and this rate can be as high as 89 % for invasive aspergillosis [12]. Furthermore, IFI significantly extends the length of stay in hospital and cause an additional economic burden. In China, the mean hospitalization cost for patients with IFI is US$17,000, which is significantly higher than that for patients without IFI (US$8,500; *p* = 0.001) [13], while in Europe, the mean total cost per patient increases to €8,360 and €15,280 for patients with possible and probable or proven invasive aspergillosis, respectively, compared with patients without invasive aspergillosis (€57,750; *p* < 0.001) [14].

Fortunately, a range of new antifungals have been developed and which demonstrated therapeutic potential over the past two decades. The echinocandins and triazoles have improved the management of IFI. The triazoles, including fluconazole, itraconazole, voriconazole, and posaconazole, have emerged as front-line drugs for the treatment and prophylaxis of IFI. Fluconazole and itraconazole were the first-generation triazoles in clinical practice. Fluconazole plays an excellent role in prophylaxis, empirical therapy, and the treatment of invasive candidiasis. Voriconazole is an effective and safe agent against the extended spectrum of fungal pathogens. Posaconazole has a similar antimicrobial spectrum to voriconazole, with additional activity against zygomycetes [15]. The echinocandins, such as caspofungin, micafungin, and anidulafungin, belong to a novel class of semisynthetic amphiphilic lipopeptides which can noncompetitively inhibit the synthesis of the β-(1,3)-D-glucan component of the cell wall of fungi. Also, the echinocandins have been demonstrated to be safe and effective in the treatment of disseminated candidiasis and invasive aspergillosis, including azole-resistant strains and biofilms [16]. The Infectious Diseases Society of America (IDSA) guidelines recommend fluconazole and echinocandins (anidulafungin, caspofungin, or micafungin) as the first-line choice for invasive candidemia [17]. Voriconazole is recommended as the initial therapy for invasive aspergillosis, while caspofungin, posaconazole, and itraconazole are alternatives [18].

The purpose of this meta-analysis was to compare the safety and efficacy of echinocandins with triazoles in the prophylaxis of fungal infection in high-risk patients and in the treatment of proven or probable fungal infections. The efficacy end points were treatment success, microbiological success, and breakthrough infection. The safety end points were drug-related adverse events (AEs), withdrawals due to AEs, and the all-cause mortality.

## Method

### Search strategies

A comprehensive search of PubMed, Embase, and the Cochrane Central Register of Controlled Trials (CENTRAL) databases from inception to July 2014 was performed. The search strategy was as follows: (echinocandin OR caspofungin OR micafungin OR anidulafungin) AND (triazole OR fluconazole OR voriconazole OR itraconazole OR posaconazole OR ravuconazole) AND random*. The results were further limited to human studies published in English. In addition, we searched for possible eligible studies in the references within the retrieved articles, as well as in review articles.

### Study selection

Two reviewers (WJ-F and XY) independently searched the literature and examined relevant randomized controlled trials (RCTs) for further assessment. A study was considered eligible if: (1) it was an RCT, (2) it included patients with proven or probable fungal infection or those at high risk of fungal infection, (3) it compared the efficacy or safety of an echinocandin with a triazole for the prophylaxis or treatment of fungal infection. Blinded and open-labeled trials were included. Trials focusing on pharmacokinetic and/or pharmacodynamic profile, dosage form evaluations, inter-echinocandin or inter-triazole comparison, topical use, pediatric or infant studies, as well as those involving combination therapy were excluded from further analysis.

### Data extraction and qualitative assessment

The same two reviewers conducted data extraction independently from eligible trials. In case of any disagreement between the reviewers, a third reviewer extracted the data and a consensus was reached. The data extraction form included the following detailed information: (1) first author, year of publication, clinical settings; (2) the number of enrolled patients and intention-to-treat (ITT); (3) antifungal agents and their doses; (4)outcomes (treatment success rate, microbiological success rate, breakthrough infection, drug-related AEs, withdrawals due to AEs, and all-cause mortality). Treatment success was defined as an endoscopy grade of 0 (zero) at the end of therapy for the treatment of esophageal candidiasis, the resolution of signs and symptoms for the treatment of other IFI, and the absence of proven, probable, or suspected systemic fungal infection through the end of prophylaxis. Microbiological success was defined as the eradication of *Candida* species and/or *Aspergillus* species from follow-up cultures in the treatment trials. Breakthrough infection was defined as any proven or probable IFI occurring during prophylaxis therapy. Efficacy outcomes (treatment success rate, microbiological success rate, breakthrough infection) were assessed in modified ITT (mITT) patients, while safety outcomes (drug-related AEs, withdrawals due to AEs, and all-cause mortality) were analyzed in the ITT population.

The methodological quality of all included trials was assessed with the Jadad scale [19], which evaluates randomization, blinding, and the number of reported dropouts or withdrawals. The score ranges were from 0 to 5 and a trial with a score higher than 2 was considered a trial of high methodological quality.

### Data analysis and statistical methods

Statistical analysis was performed using RevMan (version 5.2). Statistical heterogeneity was tested using the Cochran Q statistics generated from the χ^2^ test, and *p* < 0.10 or I^2^ > 50 % was judged to be significant. All outcomes were recorded as dichotomous data. We calculated the pooled risk ratio (RR) and 95 % confidence intervals (CIs) for all efficacy and safety outcomes using the Mantel–Haenszel fixed effects or the random-effects model according to the heterogeneity analysis. Potential publication bias was estimated by both a visual funnel plot and the Egger’s test. The Egger’s test was conducted with STATA software (version 12.0).

## Results

### Included studies and their main characteristics

**Fig. 1:**
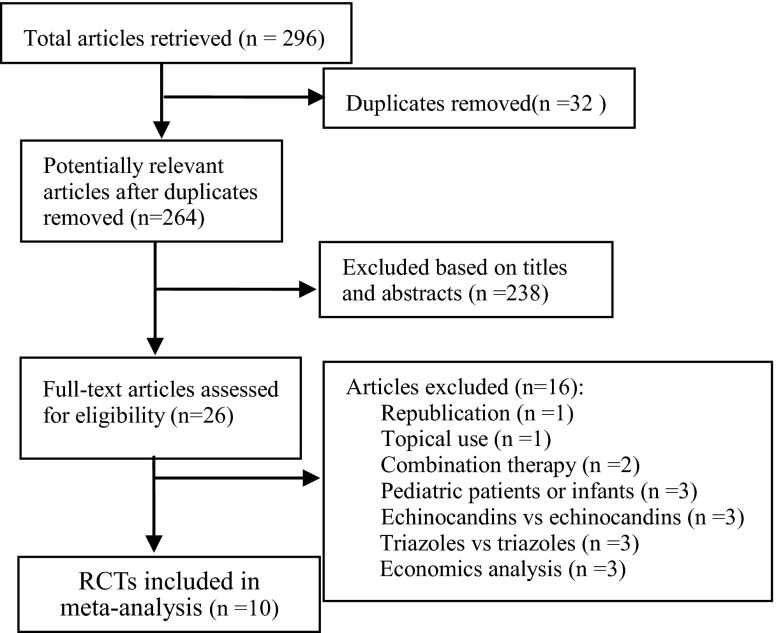
Flow diagram of the selection process for the included studies

Figure 1 shows a flow diagram of the process of identification and selection of the articles included in our study. Our literature search identified 296 potentially relevant abstracts. By screening the title and the abstract, 26 full-text articles were obtained, of which 16 were excluded due to the reasons mentioned earlier. Finally, ten studies [20–29] with a total of 2,837 patients were included in this analysis.

The main characteristics of the included trials are presented in Table 1. Among all of the included trials, six studies [20, 22–26] were related to the treatment of proven/probable fungal infections, while four studies [21, 27–29] were involved in the prophylaxis for high risk of fungal infections. One trial compared caspofungin with fluconazole [20], one trial compared caspofungin with itraconazole [21], one trial compared anidulafungin with fluconazole [22], four trials compared micafungin with fluconazole [23, 24, 27, 29], two trials compared micafungin with voriconazole [25, 26], and one trial compared micafungin with itraconazole [28]. In terms of methodology, all of the included trials were deemed to be of good quality according to the Jadad score (≥2), with nine trials being multicenter randomized and double-blinded, and the last being randomized and open-labeled.

**Table 1:** Main characteristics of the studies included in the meta-analysis

Study	Design	Patient characteristics	Interventions		No. of patients (*n*)		Jadad score
			Echinocandins	Triazoles	Enrolled	ITT (echinocandins/triazoles)	
Villanueva et al., 2002 [20]	Multicenter, double-blind RCT	Symptomatic Pts with *Candida* esophagitis	Caspofungin: i.v. 50 mg q.d.	Fluconazole: i.v. 200 mg q.d.	177	83/94	4
Mattiuzzi et al., 2006 [21]	Open-label RCT	Pts with hematologic malignancies	Caspofungin: i.v. 50 mg q.d.	Itraconazole: i.v. 200 mg q12h × 2d, followed by i.v. 200 mg q.d.	200	108/92	3
Reboli et al., 2007 [22]	Multicenter, double-blind RCT	Pts with proven invasive candidiasis	Anidulafungin: i.v. 200 mg on day 1, followed by 100 mg q.d.	Fluconazole: i.v. 800 mg on day 1, followed by 400 mg q.d.	261	131/125	3
de Wet et al., 2004 [24]	Multicenter, double-blind RCT	Pts with HIV infection and esophageal candidiasis	Micafungin: i.v. 50 mg/100 mg/150 mg q.d.	Fluconazole: i.v. 200 mg q.d.	245	185/60	3
van Burik et al., 2004 [27]	Multicenter, double-blind RCT	Pts received HSCT	Micafungin: i.v. 50 mg (or 1 mg/kg for patients weighing <50 kg) q.d.	Fluconazole: i.v. 400 mg (or 8 mg/kg for patients weighing <50 kg) q.d.	889	426/463	5
de Wet et al., 2005 [23]	Multicenter, double-blind RCT	Pts with proven *Candida* esophagitis	Micafungin: i.v. 150 mg q.d.	Fluconazole: i.v. 200 mg q.d.	523	265/258	3
Hiramatsu et al., 2008 [29]	Multicenter, open-label RCT	Pts received HSCT	Micafungin: i.v. 150 mg q.d.	Fluconazole: i.v. 200 mg q.d.	106	51/51	2
Kohno et al., 2010 [26]	Multicenter, open-label RCT	Pts with chronic pulmonary aspergillosis	Micafungin: i.v. 150~300 mg q.d.	Voriconazole: i.v. 6 mg/kg q12h on day 1, followed by 4 mg/kg q12h	107	53/54	3
Huang et al., 2012 [28]	Multicenter, open-label RCT	Pts received HSCT	Micafungin: i.v. 50 mg q.d.	Itraconazole: oral 5 mg/kg/day (in two administrations)	287	136/137	3
Shang et al., 2012 [25]	Multicenter, open-label RCT	Kidney transplant recipients with IFI	Micafungin: i.v. 100 mg/day (<60 kg) or 150 mg/day (>60 kg)	Voriconazole: i.v. 6 mg/kg q12h on day 1, followed by 4 mg/kg q12h	65	31/34	2

*Pts* patients, *RCT* randomized controlled trial, *ITT* intent-to-treat, *HSCT* hematopoietic stem cell transplantation

### Efficacy outcomes

**Fig. 2:**
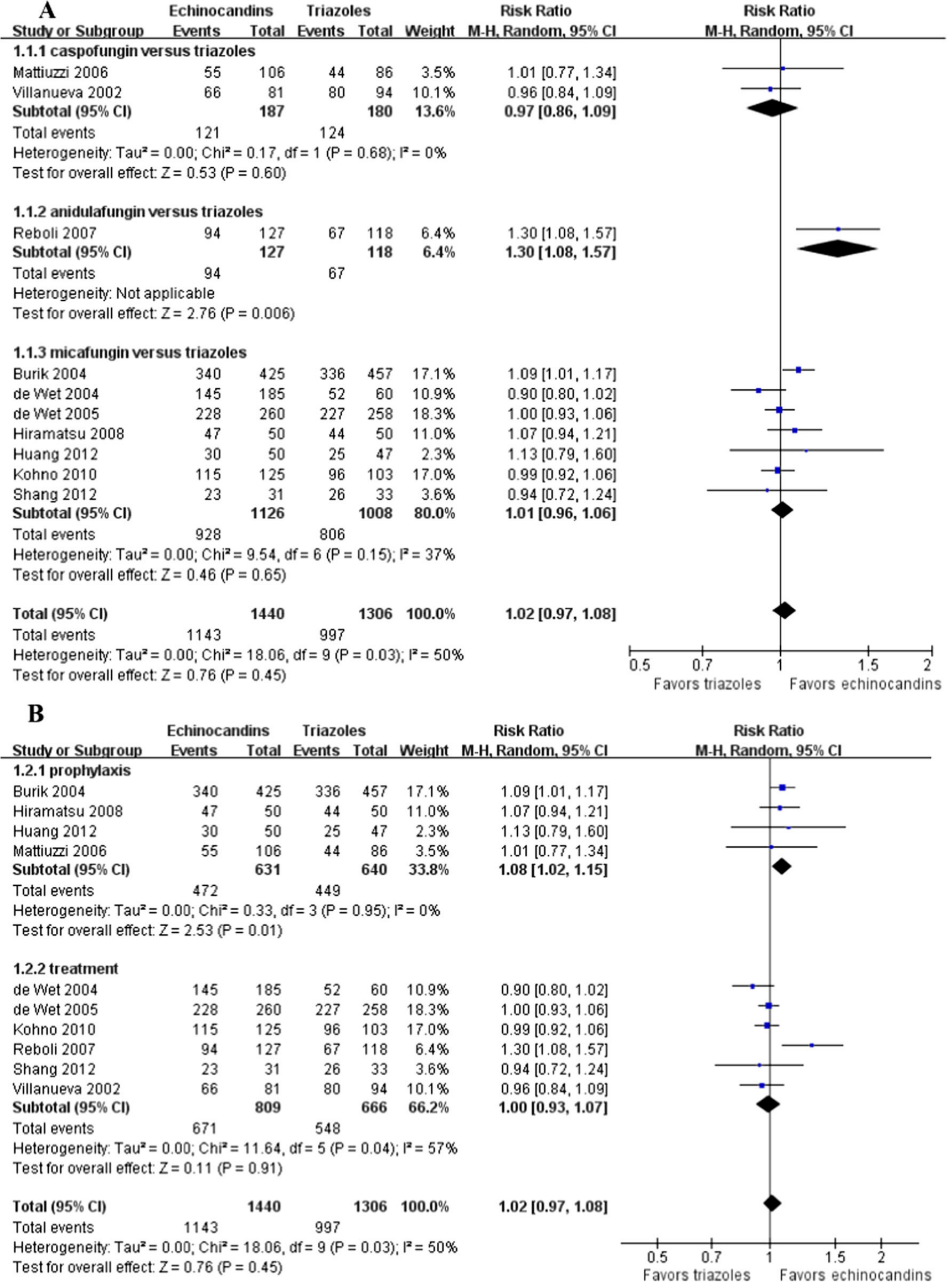
Forest plot of RRs for the treatment success rate comparing echinocandins with triazoles in the prophylaxis and treatment of fungal infections. **a** Subgroup analysis based on various echinocandins versus triazoles. **b** Subgroup analysis based on prophylaxis and treatment. *M-H* Mantel–Haenszel, *CI* confidence interval

All of the included studies reported the treatment success rate in the mITT population. The combined data suggested that there was no significant difference between echinocandins and triazoles in the treatment success rate (1,143/1,440 vs. 997/1,306; RR = 1.02; 95 % CI, 0.97–1.08; random effects), but obvious heterogeneity existed (*p* = 0.03, I^2^ = 50 %) (Fig. 2). However, the subgroup analysis showed that echinocandins were associated with significantly higher treatment success rates than triazoles for prophylaxis in patients undergoing hematologic malignancies or those who received HSCT (671/809 vs. 548/666; RR = 1.08; 95 % CI, 1.02–1.15; random effects) (Fig. 2b). For the treatment of IFI, echinocandins did not show any better treatment success than triazoles (472/631 vs. 449/640; RR = 1.02; 95 % CI, 0.97–1.08; random effects) (Fig. 2b). Meanwhile, the subgroup analysis indicated that caspofungin (121/187 vs. 124/180; RR = 0.97; 95 % CI, 0.86–1.09; random effects) or micafungin (928/1,126 vs. 806/1,008; RR = 1.01; 95 % CI, 0.96–1.06; random effects) was not superior to any of the triazoles (Fig. 2a). The Egger’s test revealed no evidence of publication bias (*p* > 0.05).

**Fig. 3:**
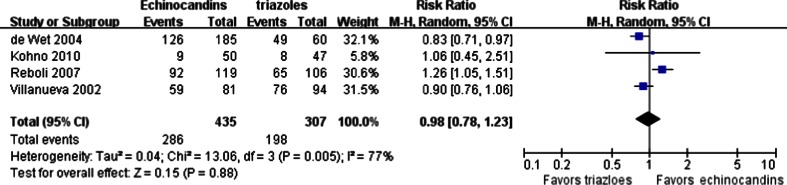
Forest plot of RRs for the microbiological success rate comparing echinocandins with triazoles in the treatment of fungal infections. *M-H* Mantel–Haenszel, *CI* confidence interval

The microbiological success rates were evaluated in four trials. *Candida* was the isolated pathogen in three trials [20, 22, 24] and *Aspergillus* in the other [26]. The pooled result showed that the microbiological success rate of echinocandins was similar to triazoles (286/435 vs. 198/307; RR = 0.98; 95 % CI, 0.78–1.23; random effects), and obvious heterogeneity was present (*p* = 0.005, I^2^ = 77 %) (Fig. 3). There was no significant publication bias detected when examined by the Egger’s test (*p* > 0.05).

**Fig. 4:**
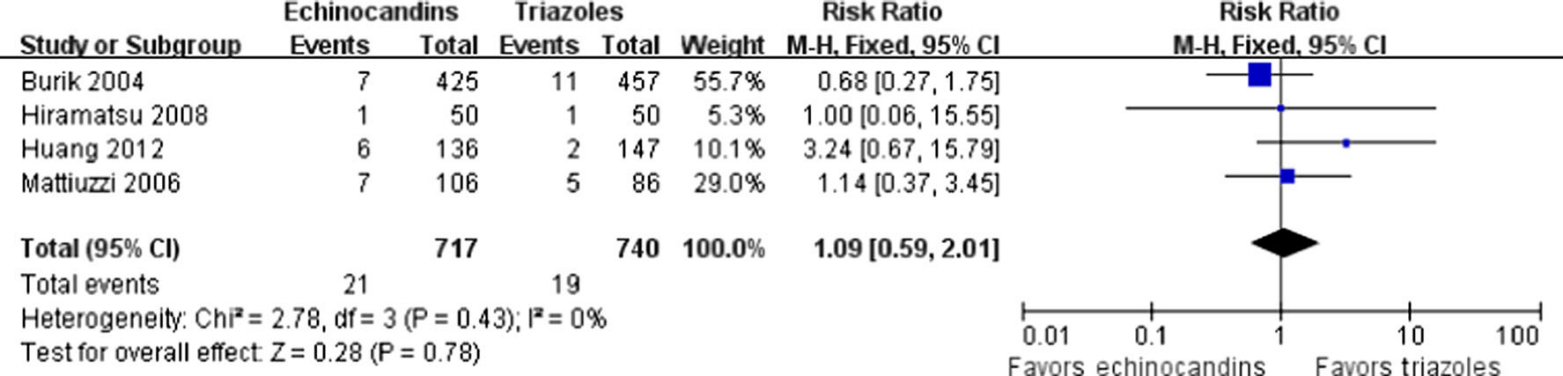
Forest plot of RRs for breakthrough infection comparing echinocandins with triazoles in the prophylaxis of fungal infections. *M-H* Mantel–Haenszel, *CI* confidence interval

Breakthrough infection after prophylaxis of fungal infections was available in four trials, in which the high-risk patients suffered from hematologic malignancies or received hematopoietic stem cell transplantation (HSCT). The pooled RR demonstrated that echinocandins were not significantly different from triazoles in breakthrough infection (21/717 vs. 19/740; RR = 1.09; 95 % CI, 0.59–2.01; fixed effects), with no statistical evidence of heterogeneity among the studies (*p* = 0.43, I^2^ = 0 %) (Fig. 4). There was no significant publication bias detected according to the Egger’s test (*p* > 0.05).

### Safety outcomes

**Fig. 5:**
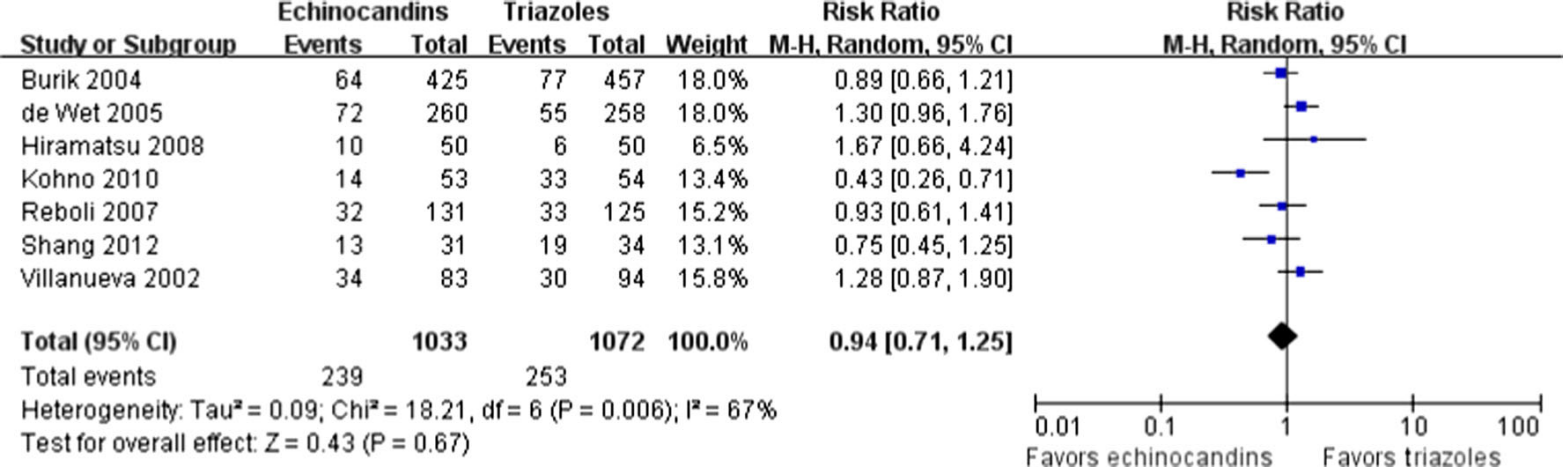
Forest plot of RRs for drug-related AEs comparing echinocandins with triazoles in the prophylaxis and treatment of fungal infections. *M-H* Mantel–Haenszel, *CI* confidence interval

The rates of AEs considered possibly or probably related to treatment were reported in seven studies. There was no significant difference between echinocandins and triazoles in drug-related AEs (239/1,033 vs. 253/1,072; RR = 0.94; 95 % CI, 0.71–1.25; random effects), and obvious heterogeneity was found (*p* = 0.006, I^2^ = 67 %) (Fig. 5). The Egger’s test revealed no evidence of publication bias (*p* > 0.05).

**Fig. 6:**
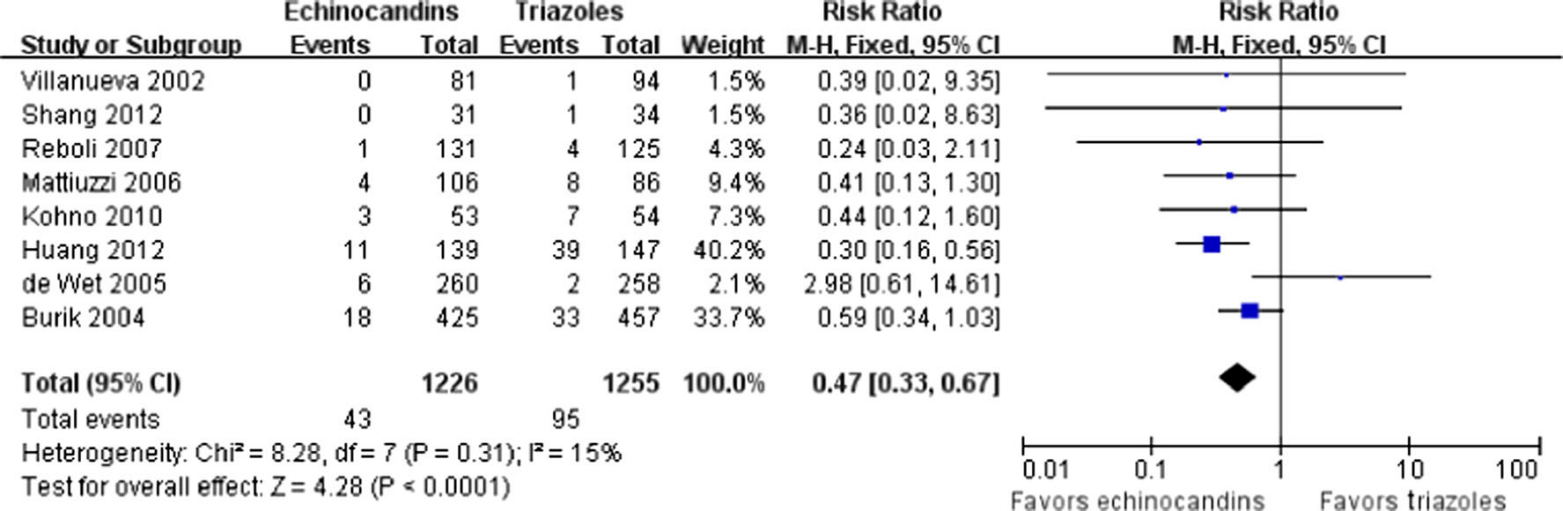
Forest plot of RRs for withdrawals due to drug-related AEs comparing echinocandins with triazoles in the prophylaxis and treatment of fungal infections. *M-H* Mantel–Haenszel, *CI* confidence interval

Eight trials showed the proportions of patients who withdrew from the trials due to drug-related AEs. A significant difference was found between the echinocandins and triazoles groups (43/1,226 vs. 95/1,255; RR = 0.47; 95 % CI, 0.33–0.67; fixed effects), and no significant heterogeneity among the studies existed (*p* = 0.31, I^2^ = 15 %) (Fig. 6). This result indicates that echinocandins may have lower rates of patient withdrawal from treatment compared to triazoles. We recorded no publication bias with the Egger’s test (*p* > 0.05).

**Fig. 7:**
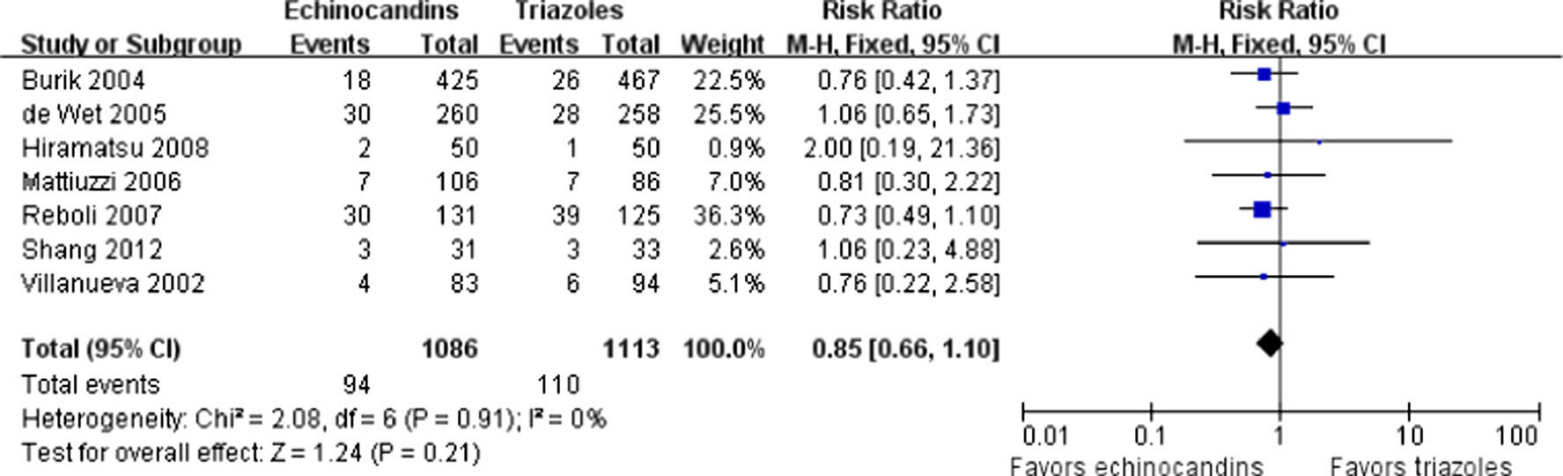
Forest plot of RRs for the all-cause mortality comparing echinocandins with triazoles in the prophylaxis and treatment of fungal infections. *M-H* Mantel–Haenszel, *CI* confidence interval

The all-cause mortality during the therapy period was available in seven trials. However, the causes of death were rarely related to the study drugs, but, instead, to the progression of infection or complication. The overall mortality in the echinocandins group was not significantly different from that in the triazoles group (94/1,086 vs. 110/1,113; RR = 0.85; 95 % CI, 0.66–1.10; fixed effects), without significant heterogeneity among the studies (*p* = 0.91, I^2^ = 0 %) (Fig. 7). There was no publication bias found based on the Egger’s test (*p* > 0.05).

## Discussion

The result of the meta-analysis by Kale-Pradhan et al. suggested that there was no significant difference in efficacy for the treatment of candidemia or invasive candidiasis between echinocandins and comparator drugs (liposomal amphotericin B, amphotericin B, and fluconazole) [30]. Caspofungin, one of the echinocandins, was also demonstrated to be as effective as other antifungal agents for the prophylaxis and treatment of fungal infections [31]. Our pooled analysis of ten RCTs with a total of 1,469 patients in the echinocandins group and 1,368 patients in the triazoles group showed similar results. It indicated that echinocandins possessed similar effects in terms of treatment success compared with triazoles in the prophylaxis and treatment of fungal infections. However, we found that echinocandins were more effective than triazoles in fungal prophylaxis by the subgroup analysis.

Micafungin is, at present, the only echinocandin approved for the prophylaxis of fungal infections in HSCT patients. Xu et al. found that micafungin was associated with a lower rate of breakthrough infection compared with fluconazole in the prophylaxis [32]. Nevertheless, we found that there was no obvious difference in breakthrough infection between echinocandins and triazoles for fungal prophylaxis. The conflict may be due to the number and type of the included trials. The trial by Hashino et al. [33] was a retrospective observational study and the trial by Hiemenz et al. [34] was associated with combination therapy, which were both excluded in our analysis. Additionally, the trial by Mattiuzzi et al. [21] associated with caspofungin was included in our review.

No differences in drug-related AEs and all-cause mortality have been found between the echinocandins and triazoles groups. Both triazoles and echinocandins have an excellent safety profile and are generally well tolerated. All triazoles have shown some degree of hepatotoxicity, ranging from mild hepatitis to cholestasis and fulminant hepatic failure [35]. Concentrations of triazoles were deemed to have no relationship with drug-related AEs. Nausea, vomiting, diarrhea, and hepatotoxicity in patients treated with triazoles may happen in the range of 5–24 % [15]. Besides, there was a 4.0–44.8 % incidence of visual changes in patients receiving voriconazole [35]. Four included studies reported, in total, seven triazoles-related serious AEs [20, 22, 26]. The serious AEs in the fluconazole arm were deep-vein thrombosis, elevated levels of hepatic enzymes, and fluconazole infusion complicated cellulitis [20, 22]. The voriconazole-related serious AEs were ventricular extra systoles, hepatic events, dizziness, and nausea. All the echinocandins patients were warned of possible hepatic dysfunction, including hepatic failure and elevated hepatic enzymes, yet the incidence was lower than that seen with the comparators [36]. In addition, rash, phlebitis, and nausea were considered to be the most frequent AEs of echinocandins. There were three echinocandins-related serious AEs reported by three included trials [22, 26], two occurred in the anidulafungin arm, including atrial fibrillation and seizures [22], and the other was disseminated intravascular coagulation because of micafungin [26].

We found that the discontinuation rate of echinocandins due to AEs was significantly lower than that of triazoles (43/1,226 vs. 95/1,255). In the triazoles groups, the discontinuation rates due to AEs of fluconazole, itraconazole, and voriconazole were about 4.3 % [20, 22, 23, 27], 20.1 % [21, 28], and 9.1 % [25, 26], respectively, while in the echinocandins groups, the rates of caspofungin, anidulafungin, and micafungin were 2.1 % [20, 21], 0.8 % [22], and 4.2 [23, 25–28], respectively. Wang et al. [37] found that the pooled risks of treatment discontinuation due to adverse reactions were 13.4 % for the amphotericin B formulations, 18.8 % for itraconazole, 2.2 % for fluconazole, 9.5 % for voriconazole, 3.8 % for caspofungin, 8.4 % for anidulafungin, and 3.6 % for micafungin.

There were several limitations in this meta-analysis. First, there was some degree of clinical heterogeneity between studies. We grouped three different echinocandins or three different triazoles together, which may lead to heterogeneity. Another potential source of heterogeneity was that we pooled together data from prophylaxis studies and treatment studies. Second, most of the included studies were associated with micafungin in the echinocandins group, only two with caspofungin, and only one with anidulafungin. As a result, the limited number of studies regarding caspofungin and anidulafungin may bias the conclusion. Most included trials were related to fluconazole in the triazoles group, while there was no RCT comparing posaconazole with echinocandins. However, Ullmann et al. found that posaconazole was superior in preventing invasive aspergillosis and reducing the rate of deaths related to fungal infections compared to fluconazole [38]. Third, the enrolled patients in the majority of trials were at high risk of mortality due to illness. When we assessed the all-cause mortality, we recognized that many patients may have died due to their original disease but not AEs. Additionally, seven trials included in this meta-analysis were industry-sponsored, a factor that may generate bias in the assessment of outcomes.

In conclusion, despite the above limitations, our results suggested that echinocandins are as effective as triazoles for the prophylaxis and treatment of patients with fungal infections. However, echinocandins may be superior to triazoles for prophylaxis only. Compared to triazoles, echinocandins were found to be equally safe regarding the incidence rate of drug-related AEs, despite the fact that withdrawals due to drug-related AEs was significantly different. Further research is needed in order to compare the efficacy and safety between posaconazole and echinocandins.
